# Investigation into longitudinal dietary behaviours and household socio-economic indicators and their association with BMI *Z*-score and fat mass in South African adolescents: the Birth to Twenty (Bt20) cohort

**DOI:** 10.1017/S1368980012003308

**Published:** 2012-07-17

**Authors:** Alison B Feeley, Eustasius Musenge, John M Pettifor, Shane A Norris

**Affiliations:** 1 MRC/WITS Developmental Pathways for Health Research Unit, School of Medicine, University of the Witwatersrand, 7 York Road, Parktown, 2193 Johannesburg, South Africa; 2 Epidemiology and Biostatistics Division, School of Public Health, Faculty of Health Sciences, University of the Witwatersrand, Johannesburg, South Africa

**Keywords:** Dietary behaviours, Socio-economic status, Obesity, Adolescents

## Abstract

**Objective:**

The present study aimed to assess the relationship between dietary habits, change in socio-economic status and BMI *Z*-score and fat mass in a cohort of South African adolescents.

**Design:**

In the longitudinal study, data were collected at ages 13, 15 and 17 years on a birth cohort who have been followed since 1990. Black participants with complete dietary habits data (breakfast consumption during the week and at weekends, snacking while watching television, eating main meal with family, lunchbox use, number of tuck shop purchases, fast-food consumption, confectionery consumption and sweetened beverage consumption) at all three ages and body composition data at age 17 years were included in the analyses. Generalized estimating equations were used to test the associations between individual longitudinal dietary habits and obesity (denoted by BMI *Z*-score and fat mass) with adjustments for change in socio-economic status between birth and age 12 years.

**Setting:**

Birth to Twenty (Bt20) study, Soweto-Johannesburg, South Africa.

**Subjects:**

Adolescents (*n* 1298; 49·7 % male).

**Results:**

In males, the multivariable analyses showed that soft drink consumption was positively associated with both BMI *Z*-score and fat mass (*P* < 0·05). Furthermore, these relationships remained the same after adjustment for socio-economic indicators (*P* < 0·05). No associations were found in females.

**Conclusions:**

Longitudinal soft drink consumption was associated with increased BMI *Z*-score and fat mass in males only. Fridge ownership at birth (a proxy for greater household disposable income in this cohort) was shown to be associated with both BMI *Z*-score and fat mass.

Since the end of the segregationist and discriminatory practices of Apartheid in 1994, South Africa has undergone profound political, social and economic transitions. Parallel to these transformations have been lifestyle changes driven by rapid rates of urbanization, from 10 % in 1990 to 56 % in 2005, especially among black South Africans^(^ ^1^ ^)^. In addition to infectious diseases and a rise in non-communicable diseases, the South African population also has the added burden of a high prevalence of HIV/AIDS and violence-related trauma^(^ ^2^ ^)^; often this collection of health challenges has been referred to as the ‘quadruple burden of disease’.

Urbanization in low- and middle-income countries drives changes in food habits and body composition and is associated with both health gains and health risks. In South Africa, between 1940 and 1992, diet among the black population shifted from a prudent pattern (>50 % carbohydrate, <30 % total fat, ∼15 % protein) to one showing a progressive increase in fat (from 16·4 % to 26·2 %) with a concurrent decrease in carbohydrate (from 69·3 % to 61·7 %)^(^ ^3^ ^)^.

The worldwide prevalence of obesity has reached alarming levels (475 million), affecting people in both high-income countries and low- and middle-income countries. Furthermore, over a billion adults are overweight^(^ ^4^ ^)^. The latest figures from South Africa show that among those aged 15 years and older the prevalence of combined overweight and obesity is 30 % among males and 55 % among females^(^ ^5^ ^)^. Of note, black women experience the greatest burden of obesity (29 %) followed by women of mixed ancestry (27 %), whites (14 %) and Indians (25 %). Among men, whites experience the highest levels (23 %), followed by mixed ancestry (15 %), Indian (11 %) and then black men (7 %). Overweight and obesity are also on the increase among younger generations and overweight has been shown to track from childhood into adulthood^(^ ^6^ ^)^. In a nationally representative study of South African children aged 1–9 years, the prevalence of combined overweight and obesity (equivalent to an adult BMI of ≥ 25·0 kg/m^2^) was 17·1 %^(^ ^7^ ^)^. It has been suggested that dietary patterns developed in childhood are maintained into adulthood, and poor dietary habits predispose individuals to obesity and related metabolic diseases later in life^(^ ^8^ ^)^.

Among the lifestyle determinants of obesity, socio-economic status (SES) has also been given attention^(^ ^9^ ^,^ ^10^ ^)^. Briefly, SES and obesity relate to each other differently depending on a country's gross national product. Among women in high-income countries a higher likelihood of obesity is found in those of lower socio-economic strata^(^ ^9^ ^)^, while in low- and middle-income countries the burden of obesity shifts towards lower SES groups as the country's gross national product increases. The shift of obesity towards women within low-SES groups seems to occur at an earlier stage of economic development than it does among men. The switch to higher rates of obesity in women of lower SES has been shown to occur when the gross national product per capita is about $US 2500^(^ ^10^ ^)^.

There are few longitudinal data and none in South Africa assessing the association between dietary behaviours developed in childhood and adolescence and overweight and obesity. Thus, using longitudinal data from urban South African adolescents at ages 13, 15, and 17 years, part of the Birth to Twenty (Bt20) cohort, we investigated dietary habits and household SES indicators and their associations with BMI *Z*-score and fat mass.

## Materials and methods

### Study population, design and sample size

Data for the present study were obtained from a longitudinal birth cohort study, the Bt20 cohort, which started in 1989^(^ ^11^ ^)^. Singleton children (*n* 3273, 78 % black participants) born between April and June 1990 and resident for at least 6 months in the Soweto-Johannesburg municipality were enrolled into the birth cohort and have been followed up almost annually between birth and 20 years of age. Attrition over the two decades has been comparatively low (30 %), mostly occurring during the children's infancy and early childhood; approximately 2300 participants remain in contact with the study^(^ ^12^ ^)^. Assessments across multiple domains have been made of children, families, households, schools and communities during the course of the study. The assessments included growth, development, psychological adjustment, physiological functioning, genetics, school performance, and sexual and reproductive health^(^ ^13^ ^)^.

**Fig. 1:**
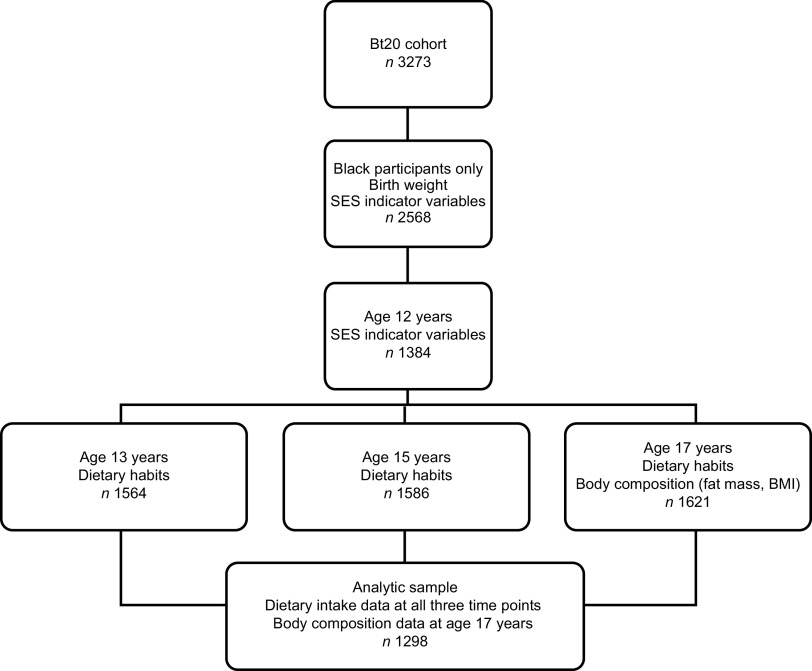
Flowchart showing measurements at the different time points for the current analysis: Birth to Twenty (Bt20) study, Soweto-Johannesburg, South Africa

Data for the current study were collected at ages 13 years (*n* 1564), 15 years (*n* 1586) and 17 years (*n* 1621). Only black participants with complete data at all three ages were included in the analysis (*n* 1298; 49·7 % male). Dietary habits data for all three ages were assessed against body composition outcomes at age 17 years (see Fig. 1).

### Dietary assessment and exposure variables

Participants completed interviewer-assisted questionnaires on dietary behaviours around food choices and eating practices, occurring in the home, school and community, that have been shown to be associated with poor nutritional outcomes^(^ ^14^ ^–^ ^22^ ^)^. The questions determined if participants engaged in a particular eating behaviour. If they did, and when appropriate, we enquired about which foods they ate (from a predetermined list) and how often they ate them in the previous week. This is similar to an unquantified FFQ approach where the frequency of certain food items consumed over the recall period is recorded. Further information on the tool's development and piloting can be found elsewhere^(^ ^23^ ^)^. We asked participants about eating behaviours in three environments of risk. (i) In the home environment, we enquired about how regularly breakfast was eaten during the week and at the weekend, how many snacks were consumed while watching television (TV) in the previous week and what snacks were eaten (e.g. crisps, bread, fruit, sweet biscuits, chocolate, popcorn, cakes, fried chips). We also enquired about how frequently participants ate their main meal with their family. (ii) In the community environment, we asked about the number (0, 1, 2, 3, 4 or >5) of fast-food items (e.g. fried chips, *vetkoek* (fried dough balls), pies, fried fish, *boerewors* (a local sausage), hot dogs, hamburger, pizza, samosa, chicken burger, filled pita), confectionery items (sweets, chocolate, doughnuts, crisps, ice cream and cake) and sweetened beverages (soft drinks and squash/cordials) consumed per week. (iii) In the school environment, we asked about the foods purchased from the tuck shop* (foods we asked about included white bread, brown bread, fruit, *pap* (mielie meal), fruit juice, milk, yoghurt, cheese, popcorn, peanuts, crisps, fried chips, pie, *vetkoek*, sweetened beverages, sweets, cake) and how many days during the previous week a lunchbox was used. Lunchbox food items we asked about included cheese, brown bread, white bread, fruit juice, fruit, sweets, crisps, sweetened beverages, yoghurt, meat, sweet biscuits, pies, milk, peanuts and *pap* (mielie meal). Over the period of data collection, meals were not provided by schools.

Dietary behaviours at each age (breakfast during the week, breakfast at the weekend, snacking while watching TV, eating main meal with family, lunchbox use, number of tuck shop purchases, fast-food consumption, confectionery consumption and sweetened beverage consumption) were categorized into binary variables, i.e. a poor eating habit (e.g. infrequent breakfast consumption or the purchase of a high number of items from the tuck shop etc.) = 1 and a ‘healthier’ eating habit (e.g. frequent breakfast consumption or the purchase of a low number of items from the tuck shop or a low number of fast food or confectionery items) = 0. See Appendix 1 for categorization of each dietary habit variable.

### Anthropometric measurements at birth and age 17 years and body composition

Birth weight was retrieved from maternity records. Birth weight *Z*-scores were calculated using the WHO 2006 growth standards^(^ ^24^ ^)^. Weight (digital scale from Dismed, USA), to the nearest 100 g, and height (stadiometer from Holtain, UK), to the nearest millimetre, were measured with participants wearing light clothing and no shoes. Body composition of the total body less the head was determined by dual-energy X-ray absorptiometry (Hologic QDR Discovery W, USA) according to standard procedures of the International Society of Clinical Densitometry (software version 11·2:3)^(^ ^25^ ^)^. For the purposes of the present study, only fat mass was used.

BMI was calculated (kg/m^2^) and internal gender-specific *Z*-scores were calculated as an alternative to using actual BMI values since BMI *Z*-scores provide a relative measure of adiposity adjusted for sex- and age-specific growth. Internal *Z*-scores were used for intra-population comparability. BMI *Z*-score was calculated as the difference between the participant's BMI and the mean BMI divided by the standard deviation of the cohort BMI for each gender. Fat mass (kg) was adjusted for height (the power coefficient was obtained from the regression analysis of fat mass (kg) *v*. height (m)) as described by Prentice *et al*.^(^ ^26^ ^)^. This measure was log-transformed to improve normality. As shown by others^(^ ^27^ ^,^ ^28^ ^)^, birth weight *Z*-score was found to be associated with the outcome measures for both genders; therefore the outcome measures were adjusted for birth weight *Z*-score by deriving the coefficient from the regression of outcome *v*. birth weight *Z*-score.

### Socio-economic indicators

SES indicators in the form of data on household durables were collected from the mother at the time of the child's birth and again when the cohort child was 12 years old. The information collected included household electricity and ownership of a TV, washing machine, landline telephone, car and fridge. Maternal education was also ascertained as an indicator of SES.

The household durables were categorized as binary variables; having a particular household durable item = 1, not having this household durable item = 0. Maternal education was categorized into those mothers who achieved a grade 10* or above = 1 and those who achieved less than this educational level = 0. Gender differences were assessed with the *χ* ^2^ test.

Confounding was assessed by performing the regression of individual household SES variables (collected at birth) *v*. outcome variables and individual dietary habits. If the household variable was significantly associated with the outcome and exposure variable, then it was considered a confounder and adjusted for in the regression models. The use of individual SES indicators resulted from preliminary exploratory analyses whereby a composite score (factor scores) of SES was derived. The score became non-significant when tested as a confounder. Upon doing factor analysis we noted that fridge ownership contributed the greatest amount (factor loadings) to the composite score. The rotated orthogonal Kaiser-varimax factor analysis showed that 23 % of the variability was explained by the first factor which had an eigenvalue of 1·39, with fridge ownership having the greatest loading of 0·97. Therefore it was decided to use individual household SES variables in the regression models.

For each SES variable a new variable was created to assess the change in SES between birth and age 12 years; that is, if the participant ‘acquired’ a particular household durable between the two time points or if they ‘never’ had that particular household durable over the two time points. These two variables were compared with the reference variable of ‘always’ having a particular household durable over the two time points. This variable was used in the multivariable analyses.

### Ethics

Ethics clearance was obtained from Witwatersrand University Committee for Research on Human Subjects (protocol number: M080320). Primary caregivers gave written informed consent for their child to participate in the research at each assessment visit and the child provided written assent. Confidentiality has been maintained by the allocation of an identification number for each participant which was used on all questionnaires.

### Statistical analysis

All statistical analyses were performed using the STATA statistical software package version 10·0 (StataCorp LP). Demographic and SES variables at birth were compared between the analytic sample (*n* 1298) and the remaining Bt20 cohort (*n* 1975).

For the analytic sample, descriptive statistics were performed for each variable, which for continuous variables were the mean and standard deviation. For categorical variables frequencies are presented and associations were assessed using the Pearson *χ* ^2^ test. Gender differences were assessed with Student's *t* test for Gaussian continuous variables.

#### Univariate and multivariable analyses

We used a generalized estimating equation (GEE) approach to fit our univariate and multivariable models; details are given in Appendix 2. For the Gaussian family-based outcomes BMI *Z*-score and adjusted fat mass, we used the identity and log link functions, respectively, with independent covariance structure. The binomial family was used for the socio-economic variables with independent covariance structures and a logit link function^(^ ^29^ ^)^. First, individual dietary habit variables were inserted individually into the statistical model; then if significantly associated (*P* < 0·05), multivariable analysis was carried out with the inclusion of the confounding household durable variables (previously assessed by univariate analysis).

## Results

### Basic characteristics and outcome variables

The analytic sample was better off at birth than the remaining Bt20 cohort in terms of having electricity (96 % *v*. 90 %, *P* < 0·001); however, the Bt20 cohort not included was better off in terms of car (32 % *v*. 25 %, *P* < 0·001) and washing machine (20 % *v*. 8 %, *P* < 0·001) ownership. No differences were found in the ownership of a TV, fridge or landline telephone or in maternal education. However, for marital status, 50 % of the Bt20 parents were either married or cohabiting compared with only 32 % of the parents of the analytic sample (*P* < 0·001).

Descriptive characteristics of the two gender groups of the analytic sample are described in Table 1. Females had a lower mean birth weight than males (*P* < 0·001). At 17 years of age, females were shorter than males (*P* < 0·001) with a similar weight; thus their mean BMI was greater than that of males (*P* < 0·001). Furthermore, mean total fat mass (less the head) was 2·4 times higher in females than in their male counterparts (*P* < 0·001). Combined overweight and obesity in this cohort was 8·1 % in males and 27·0 % in females (*P* < 0·001); this was a decrease in boys and an increase in girls, respectively, from when they were 13 years old (*P* = 0·038).

**Table 1:** Descriptive characteristics of the cohort stratified by gender: black participants, Birth to Twenty (Bt20) study, Soweto-Johannesburg, South Africa

	Males (*n* 607)		Females (*n* 616)		
Variable	Mean	sd	Mean	sd	*P* value
Birth weight (g)	3128·7	504·8	3012·8	491·6	<0·001
Birth weight *Z*-score†	−0·52	1·1	−0·54	1·1	0·639
Age (years)	17·7	0·3	17·7	0·3	1·000
Height (cm)	170·6	7·9	159·7	6·0	<0·001
Weight (kg)	59·3	9·7	58·5	12·2	0·253
BMI (kg/m^2^)	20·4	3·2	22·9	4·4	<0·001
Total fat mass (kg) less head	7·7	4·8∥	18·5	7·6††	<0·001
Overweight/obese‡ (*n*, %)	43	8·1	152	27	<0·001
Age (years)	13·7	0·2	13·7	0·2	1·000
Height (cm)	154·3	8·5¶	155·7	6.2‡‡	<0·001
Weight (kg)	44·5	9·8	50·0	11·6	<0·001
BMI (kg/m^2^)	18·6	3·2	20·6	4·3	<0·001
Overweight/obese§ (*n*, %)	52	9·8	115	20·5	0·038

†WHO 2006 growth standards^(^ ^24^ ^)^.

‡Using the International Obesity Taskforce cut-offs for 17·5-year-olds^(^ ^52^ ^)^.

§Using the International Obesity Taskforce cut-offs for 13·5-year-olds^(^ ^52^ ^)^.

∥*n* 592.

¶*n* 529.

††*n* 594.

‡‡*n* 561.

Table 2 shows dietary habits and eating practices, stratified by gender, at ages 13, 15 and 17 years. In both boys and girls, irregular breakfast (both weekday and weekend) consumption increased with age, with girls consistently skipping breakfast more often than boys (*P* < 0·05). Between 30 and 40 % of the cohort ate their main meal with their family infrequently (never/some days) throughout the follow-up period. Over two-thirds consumed fast foods and sweetened beverages on three or more occasions per week. Over two-thirds consumed confectionery on seven or more occasions per week, with girls consuming them more than boys at ages 15 and 17 years (*P* < 0·05). Generally lunchbox usage was low (5–20 %), with girls using them more regularly than boys at all ages (*P* < 0·05). Between 50 and 70 % of participants purchased ten or more tuck shop items per week.

**Table 2:** Dietary habits variables stratified by age and gender: black participants, Birth to Twenty (Bt20) study, Soweto-Johannesburg, South Africa

	Age 13 years				Age 15 years				Age 17 years			
	Males (*n* 645)		Females (*n* 653)		Males (*n* 645)		Females (*n* 653)		Males (*n* 645)		Females (*n* 653)	
Exposure	*n*	%	*n*	%	*n*	%	*n*	%	*n*	%	*n*	%
Home												
Irregular breakfast weekday (<2/week)	136	21·1	188***	28·8	176	27·3	304***	46·6	193	29·9	268***	41·0
Irregular breakfast weekend (1 d)	79	12·3	91	13·9	107	16·6	148**	22·7	220	34·1	180	27·6
Irregularly eat with family (never/some days per week)	220	34·2	244	37·4	222	34·6	254	38·9	269	41·7	257	39·4
TV snacks (>3/week)	353	54·7	400**	61·2	329	51·0	395*	60·5	380	58·9	450***	68·9
Community												
Fast foods (>3/week)	438	67·9	454	69·5	440	68·2	422	64·6	415	64·3	395	60·5
Confectionery (>7/week)	386	59·8	422	64·6	415	64·3	495***	75·8	380	58·9	435**	66·6
Sweetened beverages (>2/week)	431	66·8	451	69·1	452	70·1	466	71·4	431	66·8	450	68·9
School												
Lunchbox (≤2/week)	539	84·3	519*	79·7	581	92·1	531***	85·5	618	95·8	563***	86·2
Tuck shop purchases (>10/week)	461	64·5	452	69·2	438	49·9	445	68·2	434	67·3	427	65·4

TV, television.

Significant difference between genders: *P < 0·05, **P < 0·01, ***P < 0·001.

There were no gender differences in household SES indicators or maternal education at the birth of the cohort participants. At that time, most of the cohort families had electricity in the home (96 %) and a TV (80 %) and a fridge (76 %). A smaller proportion owned a landline telephone (<58 %), a car (<26 %) or a washing machine (8 %). Nearly half the mothers (45 %) had schooling to grade 10 or above, with only 7 % of this group having had post-school education.

### Univariate models

#### Dietary habits and practices

Univariate GEE between body composition outcome measures at age 17 years and each longitudinal dietary habit and practice variable were assessed. All GEE models were stratified by gender, since gender differences were shown for the outcome and exposure variables.

Among males, the univariate analyses showed that longitudinal sweetened beverage consumption was positively associated with both BMI *Z*-score (*β* = 0·050, 95 % CI 0·014, 0·085; *P* = 0·007) and fat mass (*β* = 0·035, 95 % CI 0·007, 0·063; *P* = 0·015), while infrequent consumption of the main family meal was negatively associated with fat mass (*β* = −0·06, 95 % CI −0·052, −0·001; *P* = 0·038).

Among females, positive associations were found between irregular weekend breakfast consumption and BMI *Z*-score (*β* = 0·044, 95 % CI 0·0135, 0·075; *P* = 0·005) and fat mass (*β* = 0·030, 95 % CI 0·000, 0·060; *P* = 0·047). As with boys, infrequent consumption of the main family meal was negatively associated with fat mass (*β* = −0·026, 95 % CI −0·052, −0·001; *P* = 0·038).

#### Confounders

The regressions of household durable variables *v*. the exposure and outcome variables were performed separately (Table 3). Among males only, fridge ownership was positively associated with both BMI *Z*-score and fat mass and with one exposure variable, soft drink consumption (*P* < 0·05). No other SES variables were associated with either exposure or outcome variables, for either gender.

**Table 3:** Significant associations (regression coefficient *β*, 95 % confidence interval) estimated by GEE in univariate analyses regarding the SES indicators of household durables and maternal education in relation to individual dietary habits and eating practices and outcome variables (BMI *Z*-score and fat mass), stratified by gender: black participants, Birth to Twenty (Bt20) study, Soweto-Johannesburg, South Africa

	Fridge		TV		Car		Electricity		Washing machine		Landline telephone		Maternal education	
	Males	Females	Males	Females	Males	Females	Males	Females	Males	Females	Males	Females	Male	Females
Irregular breakfast weekday														
*β*	X	X	X	X	X	X	X	0·122*	X	−0·010**	X	−0·061**	X	X
95 % CI								0·005, 0·239		−0·182, −0·023		−0·107, −0·016		
Irregular breakfast weekend														
*β*	X	X	X	X	X	X	X	0·104***	X	X	X	X	X	X
95 % CI								0·004, 0·203						
TV snacking														
*β*	0·108***	0·067**	0·056***	X	X	X	0·127***	X	X	X	X	0·058**	X	0·062***
95 % CI	0·055, 0·160	0·017, 0·118	0·001, 0·112				0·012, 0·242					0·014, 0·103		0·019, 0·104
Irregularly eat with family														
*β*	X	0·055***	X	X	X	0·064**	X	X	X	X	X	X	X	X
95 % CI		0·003, 0·106				0·015, 0·114								
Fast food														
*β*	X	X	0·055*	−0·056*	0·077**	X	X	X	0·086**	X	X	X	X	0·052**
95 % CI			0·002, 0·108	−0·110, −0·002	0·027, 0·128				0·009, 0·167					0.010, 0·095
Confectionery														
*β*	X	X	X	X	X	X	0·164**	X	X	X	−0·050***	X	X	X
95 % CI							0·052, 0·276				−0·096, −0·004			
Sweetened beverages														
*β*	0·088***	0·057**	X	X	0·059**	X	0·138**	X	0·120**	X	X	X	X	0·103***
95 % CI	0·039, 0·137	0·009, 0·106			0·010, 0·109		0·031, 0·246		0·042, 0·195					0·063, 0·144
Lunchboxes														
*β*	X	X	X	X	X	X	X	X	−0·078***	X	X	−0·042**	−0·035***	X
95 % CI									−0·126, −0·031			−0·078, −0·006	−0·061, 0·009	
Tuck shop														
*β*	X	X	X	X	X	X	0·167**	X	X	X	X	X	X	X
95 % CI							0·058, 0·276							
BMI *Z*-score														
*β*	0·079***	X	X	X	X	X	X	X	X	0·069**	X	X	X	−0·031*
95 % CI	0·044, 0·113									0·196, 0·119				−0·058, −0·004
Fat mass														
*β*	0064***	X	X	X	X	X	X	X	X	0·095***	0·062***	X	X	X
95 % CI	0·340, 0·094									0·045, 0·145	0·036, 0·089			

GEE, generalized estimating equations; SES, socio-economic status; TV, television.

Significance of association: *P < 0·05, **P < 0·01, ***P < 0·001.

#### Birth weight Z-score

Linear regression showed that birth weight *Z*-score was positively associated with BMI *Z*-score and fat mass at 17 years of age (*P* < 0·001) for both genders (Table 4). Therefore in the multivariable analyses each outcome variable was adjusted for birth weight *Z*-score.

**Table 4:** Association (regression coefficient *β*, 95 % confidence interval) of birth weight *Z*-score with BMI *Z*-score and fat mass (unadjusted and adjusted for height) at age 17 years, stratified by gender: black participants, Birth to Twenty (Bt20) study, Soweto-Johannesburg, South Africa

	Unadjusted for height		Adjusted for height	
	*β*	95 % CI	*β*	95 % CI
BMI *Z*-score				
Males	0·150***	0·084, 0·216	–	–
Females	0·128***	0·063, 0·193	–	–
Fat mass				
Males	0·702***	0·354, 1·049	0·268***	0·119, 0·417
Females	1·092***	0·577, 1·607	0·772***	0·382, 1·161

Significance of association: ***P < 0·001.

### Multivariable models

Multivariable GEE were conducted separately for both outcome measures (BMI *Z*-score and fat mass) and for all exposures and confounders.

Only sweetened beverage consumption was positively and significantly associated with BMI *Z*-score in the unadjusted model. Furthermore, after adjustment for confounders (household assets, in this case fridge ownership), the association between sweetened beverage consumption and BMI *Z*-score remained (*P* < 0·05; see Table 5). For fat mass, sweetened beverage consumption was also positively and significantly associated in the unadjusted model, and after adjusting for confounding the relationship remained the same (*P* < 0·001; see Table 5).

**Table 5:** Associations (regression coefficient *β*, 95 % confidence interval) of BMI *Z*-score and fat mass with sweetened beverage consumption in unadjusted and final multivariable models among males: black participants, Birth to Twenty (Bt20) study, Soweto-Johannesburg, South Africa

	Unadjusted model		Adjusted models	
	*β*	95 % CI	Adj. *β*	95 % CI
BMI *Z*-score				
Sweetened beverages	0·022***	0·006, 0·037	0·044**	0·022, 0·067
Fridge, never			−0·095**	−0·152, −0·039
Fridge, acquired			−0·026	−0·056, 0·004
Fat mass				
Sweetened beverages	0·016***	0·005, 0·030	0·018*	0·002, 0·036
Fridge, never			−0·040	−0·083, 0·002
Fridge, acquired			−0·034***	−0·057, −0·011

Significance of association: *P < 0·05, **P < 0·01, ***P < 0·001.

## Discussion

The aim of the present study was to investigate adolescent dietary behaviours and their association with BMI *Z*-score and fat mass. Among males only, in both the unadjusted and adjusted models we found that sweetened beverage consumption was positively associated with both BMI *Z*-score and fat mass (*P* < 0·001). These findings reflect the importance of sweetened beverage consumption in terms of the quantity and range consumed (regarding their energy content) and their relationship with obesity. In this cohort, 76 % of participants owned a fridge which differentiates them from poorer households. Other studies have shown the use of multiple individual measures of SES when assessing children's nutritional status^(^ ^30^ ^)^. Furthermore, in a sub-study of the Bt20 cohort, individual household durable variables were shown to act as a proxies for higher SES^(^ ^31^ ^)^. In this cohort, males from families with higher disposable household income (denoted by fridge ownership) at birth were more predisposed to overweight (denoted by BMI *Z*-score and fat mass) than those from families with lower income.

Other studies have found an association between adolescent soft drink consumption and obesity, both in low- and middle-income countries and high-income countries. Cross-sectional associations have been found for soft drink consumption and obesity in Saudi boys^(^ ^32^ ^)^ and both Jamaican males and females^(^ ^33^ ^)^. Unlike cross-sectional studies, longitudinal studies are able to account for the temporal criteria of causality in that repeated observations are possible. However, whereas some US longitudinal studies have found an association between soft drink consumption and obesity^(^ ^34^ ^)^, others have not^(^ ^35^ ^)^. Perhaps the equivocal findings relate to the concept that soft drinks may be a marker for other dietary factors or other lifestyle factors that are associated with obesity, or due to study design and questionnaire nuances, including the definition of a soft drink.

Research into specific dietary habits associated with poor diet quality and obesity risk among adolescents has focused on breakfast skipping, snack behaviours (including the influence of TV viewing on snacking), food intake at school, eating the main meal with the family, fast-food intake and sweetened beverage consumption^(^ ^36^ ^–^ ^38^ ^)^. While a number of cross-sectional analyses have shown positive associations between eating behaviours (breakfast skipping, fast-food intake, soft drink consumption and eating the main meal with the family) and obesity, these relationships have been attenuated and become statistically non-significant in some longitudinal analyses^(^ ^39^ ^,^ ^40^ ^)^. On the other hand, other longitudinal studies have shown positive relationships with obesity, namely with fast food-intake and soft drink consumption and breakfast skipping, among adult populations^(^ ^41^ ^)^ and adolescent populations^(^ ^42^ ^,^ ^43^ ^)^.

The longitudinal dietary patterns of the present Bt20 cohort show that both boys and girls have increasingly irregular breakfast (weekday and weekend) consumption with age, with girls consistently skipping breakfast more often than boys, findings corroborated by other studies in developing countries^(^ ^44^ ^)^. Eating the main meal with the family decreased slightly when participants were 17 years old. In the USA, older adolescents eat with their families less frequently than younger adolescents; furthermore, advancing age has been associated with irregular family meal consumption and poorer diet quality^(^ ^45^ ^)^. Although cross-sectional analyses have shown that the family meal has a protective effect against obesity, longitudinal analyses have not confirmed this^(^ ^37^ ^)^.

Increased TV viewing has been shown to be associated with reduced fruit and vegetable consumption, and more snacking^(^ ^36^ ^)^. One study found that every additional hour of TV viewed equated to an additional intake of 653 kJ/d^(^ ^46^ ^)^. In our study, the number of snacks eaten while watching TV increased with age and girls consistently consumed them more frequently than boys (*P* < 0·01).

US ecological data have shown that snacking (including confectionery) in adolescents has increased between 1977 and 2006, with about three snacks eaten per day which account for up to 27 % of total energy intake^(^ ^38^ ^)^. In our cohort a higher proportion of girls consumed confectionery seven or more times weekly than boys at all ages.

It is thought that bringing food from home to school is healthier than purchasing items available at the school tuck shop but in the Bt20 cohort lunchbox usage was low (5–20 %) across all ages, with boys using them less regularly than girls (*P* < 0·05). However we did show that of those who took lunchboxes to school, the five most popular items were relatively healthy (brown and white bread, cheese, fruit and fruit juice)^(^ ^23^ ^)^.

SES can influence dietary intake and eating behaviours through purchasing of foods. In a developing environment such as Soweto, those in higher income strata have a greater disposable income to purchase relatively expensive fast foods and snacks. However what is unique about this environment is the access to informal food vendors, which makes such energy-dense foods also available to those in poorer income strata. We have observed the sale of cheap snacks and fast foods both in poorer rural and more affluent urban environments^(^ ^47^ ^,^ ^48^ ^)^.

The prevalence of combined overweight and obesity was significantly higher in females than males in the present study, which is consistent with other South African research^(^ ^49^ ^)^. However, the data show that dietary patterns are not mediated by SES among this female group, which is contrary to other research undertaken in high-income countries and low- and middle-income countries^(^ ^9^ ^,^ ^10^ ^)^. Perhaps the lack of evidence of a relationship between SES and obesity among females might reflect the choice of indicators used in the research; for example, it has been demonstrated – in men at least – that the SES–obesity relationship depends on the indicator under assessment^(^ ^9^ ^)^. Another possible reason why no associations were found among females in the Bt20 cohort is because we are witnessing a change in the social patterning of overweight/obesity, as suggested by Monteiro *et al*.^(^ ^10^ ^)^. For example, in some countries, for certain SES indicators the association with obesity was more often negative than positive, suggesting that the social patterning of overweight is possibly undergoing transition in middle-income countries^(^ ^10^ ^)^ and reflecting that of developed countries. Another possibility is that the cohort did not reflect a very wide distribution of SES since most can be defined as poor as compared with high-income countries.

An alternative hypothesis suggests that the greater prevalence of obesity among females (both in this population and others in South Africa^(^ ^5^ ^,^ ^49^ ^)^) relates to nutritional programming *in utero*, whereby a relationship exists between the environment during critical windows of development and the progression of disease in adult life^(^ ^50^ ^)^. Studies have shown that perinatal nutritional deficits predispose adult offspring to increased fat accumulation and other metabolic outcomes. Another explanation for our lack of a finding in girls might relate to physical activity. Other South African research has reported declines in physical activity with girls exercising less often than boys^(^ ^51^ ^)^.

A limitation of the present study is that its findings cannot be extrapolated to other subgroups in South Africa because we assessed only black South African adolescents in Soweto, reflecting a particular social stratum. Another limitation is that total energy intake or energy expenditure was not investigated.

## Conclusion

In summary, we showed that longitudinal sweetened beverage consumption was positively and significantly associated with both BMI *Z*-score and fat mass at age 17 years among males. Furthermore, fridge ownership at birth (a proxy for greater household disposable income in the Bt20 cohort) was shown to be associated with BMI *Z*-score and fat mass.

## Footnotes

* Which may have been a food vendor selling foods within or outside the school grounds.

* Equivalent to grade 11 in the USA and the legal point of exiting the education system in South Africa.

## Appendix 1

### Categorization of dietary habit variables

Continuous exposure variables were categorized into binary variables:

**Table:**

	Positive eating behaviour (0)			Negative eating behaviour (1)		
Exposure variable		%		%
No. of TV snacks (TV)	≤3	35·5	>3	64·5
No. of fast food (FF) items	≤3	38·5	>3	61·5
No. of confectionery (conf) items	≤7	37·0	>7	63·0
No. of sweetened beverages (SB)	≤1	31·6	>2	68·4
No. of tuck shop purchases (TS)	≤9	36·0	>10	64·0

Coding for categorical variables:

**Table:**

	Positive eating behaviour (0)	Negative eating behaviour (1)
Breakfast weekday (brkd)	3+/week	0–2/week
Breakfast weekend (brkw)	2 during weekend	0–1 during the weekend
Eat main meal with family (EatF)	Most days or every day	Never or some days
Lunchbox (LB)	3–5/week	0–2/week

## Appendix 2

### The generalized estimating equation model structure

The data for our study were longitudinal since they were collected at ages 13, 15 and 17 years. As such, the observations are correlated at the individual level. We thus employed GEE, which are an extension of the generalized linear models (GLM) that can handle correlation in data. Our model, which follows from the exponential family of distributions GLM introduced, has the link form:

where  is a *p* × 1 vector of covariates for the *i*th subject,  is a *p* × 1 vector of regression coefficients, g(·) is the link function which can take any form of the exponential family, *Y_i_* is the outcome of the *i*th subject and *μ_i_* is the mean of *Y_i_* (or expectation of *Y_i_* = *E*[*Y_i_*])^(^ ^29^ ^)^.

In the GEE extension of this model for repeated measures, we model the average response for observations sharing the same covariates (marginal expectation) as:

where  is a *p* × 1 vector of covariates for the *i*th subject (*i* = 1,2,…,*n*) at the *j*th outcome (*j* = 1,2,…,*t*),  is a *p* × 1 vector of regression coefficients, g(·) is the link function which can take any distributional form and *Y_ij_* is the outcome of the *i*th subject at the *j*th outcome whose mean and variance are characterized as the GLM^(^ ^53^ ^)^.

The correlated observations have a certain working correlation matrix, **R**(**α**), of the forms: independent, exchangeable, unstructured, time series auto-regressive orders, user defined and many others. Assuming no missing data, the *t* × *t* covariance structure for *Y_i_* is the following, with **A** *_i_* a matrix of variance functions and Φ the GLM dispersion parameter:

In this paper we fit our models using three link functions: the identity function  for BMI *Z*-score, the log link function  for fat mass adjusted for height and the logit link function  for the binary socio-economic variables. Our families of distributions were the Gaussian and the binomial. The working correlation was the identity matrix for the independent covariance structure.
